# Effectiveness of a Simultaneous rHVT-F(ND) and rHVT-H5(AI) Vaccination of Day-Old Chickens and the Influence of NDV- and AIV-Specific MDA on Immune Response and Conferred Protection

**DOI:** 10.3390/vaccines8030536

**Published:** 2020-09-16

**Authors:** Fabienne Rauw, Eva Ngabirano, Yannick Gardin, Vilmos Palya, Bénédicte Lambrecht

**Affiliations:** 1 Sciensano, Avian Virology and Immunology Service, Groeselenberg 99, 1180 Brussels, Belgium; eva.ngabirano@sciensano.be (E.N.); benedicte.lambrecht@sciensano.be (B.L.); 2 CEVA Santé Animale, Avenue de la Ballastière 10, 33 500 Libourne, France; yannick.gardin@orange.fr; 3 CEVA Phylaxia, Szállás utca 5, 1107 Budapest, Hungary; vilmos.palya@ceva.com

**Keywords:** chicken, Newcastle disease, avian influenza, recombinant vaccine, turkey herpesvirus, protection

## Abstract

The recombinant herpesvirus of turkey (rHVT) vaccines targeting Newcastle disease (ND) and H5Nx avian influenza (AI) have been demonstrated efficient in chickens when used individually at day-old. Given the practical field constraints associated with administering two vaccines separately and in the absence of a currently available bivalent rHVT vector vaccine expressing both F(ND) and H5(AI) antigens, the aim of this study was to investigate whether interference occurs between the two vaccines when simultaneously administered in a single shot. The studies have been designed to determine (i) the ND and AI-specific protection and antibody response conferred by these vaccines inoculated alone or in combination at day-old, (ii) the influence of maternally-derived antibodies (MDA), and (iii) the potential interference between the two vaccine. Our results demonstrate that their combined administration is efficient to protect chickens against clinical signs of velogenic Newcastle disease virus (vNDV) and H5-highly pathogenic avian influenza virus (HPAIV) infections. Viral shedding following co-vaccination is also markedly reduced, while slightly lower NDV- and AIV-specific antibody responses are observed. NDV- and AIV-specific MDA show negative effects on the onset of the specific antibody responses. However, if AIV-specific MDA reduce the protection against H5-HPAIV induced by rHVT-H5(AI) vaccine, it was not observed for ND.

## 1. Introduction

Newcastle disease virus (NDV) and avian influenza virus (AIV) are two of the most important viral pathogens currently affecting the poultry industry worldwide. Vaccinations have been shown to protect poultry against clinical signs and mortality. They can also markedly reduce virus shedding, thereby reducing viral transmission and inducing higher resistance to infection. Currently, herpesvirus of turkey (HVT), mainly the FC126 strain, is widely and successfully used as a vector for the production of recombinant HVT (rHVT) vaccines [1]. This success is related to (i) the safety and genetic stability of the HVT vector, (ii) its early administration either in vivo or at day-old without adverse effects on normal hatchability or survival rates of vaccinated chicks, (iii) its lower sensitivity to interference from maternally-derived antibodies (MDA) when injected in cell-associated form, and (iv) its potential for long-term induction of protective immunity against targeted pathogens with reactivation of the HVT vector from latency [2].

Several laboratories and field trials have demonstrated the efficacy induced by the individually administration of rHVT vaccines targeting Newcastle disease (ND) [3,4] and H5Nx avian influenza (AI) [5,6]. Given the practical field constraints associated with administering two vaccines separately and particular disease conditions, previous studies have shown that combinations of rHVT vaccines targeting ND, infectious bursal disease, and infectious laryngotracheitis reduce the efficacy of the vaccines administered [7,8]. In this context, and in the absence of a currently available bivalent rHVT vector vaccine expressing both F(ND) and H5(AI) antigens, we investigated the possible effectiveness in the poultry sector of rHVT-F(ND) and rHVT-H5(AI) vaccines administrated simultaneously in a single shot to day-old chickens. The studies have been designed to determine (i) the ND and AI-specific protection and antibody response conferred by ND and AI vaccination schedule using the rHVT-F(ND) and rHVT-H5(AI) vaccines in combination at day-old, (ii) the influence of MDA, and (iii) the interference between rHVT-F(ND) and rHVT-H5(AI) vaccines.

## 2. Materials and Methods

### 2.1. Chickens

Experiments were initially conducted with specific-pathogen-free (SPF) White Leghorn (WL) layer chickens hatched from eggs provided by Prophyl Ltd. (Mohacs, Hungary). Lohmann Brown Light (LBL) layer chickens (not SPF) with and without NDV- or AIV-specific MDA (MDA(+) and MDA(–), respectively) were also used to determine the impact of MDA on the vaccination effectiveness. Day-old MDA(–) LBL layer chickens were obtained from commercial breeder chickens which were previously vaccinated against Marek’s disease (MD) with Rispens and HVT vaccines. However, they had not received any type of ND or AI vaccines. These breeder chickens were acquired in Sweden, transported to Prophyl Ltd. (Hungary), and kept in isolated facilities. Day-old MDA(+) chicks were obtained after ND and AI vaccination of a subset of the imported breeders. The vaccination program included (i) live attenuated ND vaccines (Avinew, Merial, and Clone 30, MSD) during the growing period, and (ii) inactivated AI (Reassortant avian influenza virus vaccine, inactivated H5N1 subtype, Re-1 strain, HVRI, China) and ND vaccines (Gallimune 403, Merial and Cevac ND EDS K, Ceva) in a prime-boost regime.

After hatching, all birds were kept in biosecurity level 3 (BSL-3) isolators. Animal experiments were conducted under the authorization and supervision of the Biosafety and Bioethics Committees at Sciensano (Brussels, Belgium: bioethics authorization no. 101105-01; biosafety authorization no. FARAU 06.06.11, and no. FARAU 12.01.12) and at Ceva Phylaxia (Budapest, Hungary: bioethics authorizations no. BA01/2010-3 and no. BA/2005/2012; biosafety authorization no. 02.1/165/7/2011) according to national and European regulations.

### 2.2. Vaccines and Challenge Strains

Both commercial cryopreserved cell-associated rHVT expressing either native F protein (rHVT-F(ND)) or H5 protein (rHVT-H5(AI)) from the H5N1 clade 2.2 H5-HPAIV *A/Swan/Hungary/4999/2006* strain have been described previously [9,10].

Each vaccine was diluted in corresponding vaccine diluent (Ceva-Biomune, Lenexa, KS, USA) to obtain one dose of 4000 plaque forming units (PFU) in 200 µL. For simultaneous application of rHVT-F(ND) and rHVT-H5(AI), a 4000 PFU dose of each vaccine was diluted in a final volume of 200 µL of diluent. Each individual and combined vaccine dose was administered as a subcutaneous inoculation in the neck of day-old chickens.

The *D1524/1/1,2/MY/10* velogenic Newcastle disease virus (vNDV) strain used for challenge was isolated in Malaysia in 2010 and is classified as an NDV genotype VII strain [4]. Animals were challenged with 100 µL of inoculum containing 10^5^ median embryo infectious dose (EID_50_) vNDV administered via the intra-nasal route, with 50 µL applied to each nostril. The H5N1 *A/Chicken/1709-6/2008* highly pathogenic avian influenza virus (HPAIV) strain used for AI challenge originated in Egypt and belongs to genetic clade 2.2.1 [10,11]. Animals were challenged with 100 µL of inoculum containing 10^6^ EID_50_ H5-HPAI via the oculo-nasal route, with 50 µL applied to the eye and 50 µL to the nostril.

### 2.3. Experimental Design

In the first trial, day-old SPF WL layer chickens were assigned to four groups to receive rHVT-F(ND) (batch no. 372-207) (ND vaccination), rHVT-H5(AI) (batch no. 395-004) (AI vaccination), both vaccines (AI/ND vaccination), or no vaccine (unvaccinated controls). In ND experiments conducted at Ceva Phylaxia, serum was obtained from 20 vaccinated chickens from each group and from 10 unvaccinated control birds at 2, 3, 4, 6, and 8 weeks of age. Twenty vaccinated birds from each group and 10 unvaccinated chickens were individually identified and challenged with vNDV at 3 and 8 weeks of age. In the AI experiments conducted at Sciensano, serum was collected from 5 chickens per group at weekly intervals from 2 weeks to 8 weeks of age. At 4 weeks and 8 weeks, 10 birds from each group were individually identified and challenged with H5-HPAIV. During a 2-week post-challenge observation period, chickens exhibiting clinical signs (prostration, diarrhea, respiratory distress), neurological signs (loss of balance, torticollis, lack of coordination), or mortality were considered to be unprotected. Oro-nasal and cloacal swabs were collected 3 days and 7 days after vNDV challenge [4], while tracheal and cloacal swabs were collected 2, 4, 7, and 10 days after H5-HPAIV challenge [11].

In the second trial, MDA(–) and MDA(+) day-old LBL layer chickens were assigned to four groups according to their vaccination schedule, similar to trial I. Briefly, ND experiments were conducted at Ceva Phylaxia with serum collected at 4, 7 and 10 weeks of age from 20 vaccinated chickens from each group and from 10 unvaccinated control chickens. Twenty vaccinated birds from each group and 10 unvaccinated chickens were individually identified and challenged with vNDV at 4, 7, and 10 weeks of age. In AI experiments conducted at Sciensano, serum was collected from 5 chickens per group at 4, 7, and 10 weeks of age. At the same time points, 10 birds from each group were individually identified and challenged with H5-HPAIV. Chickens were monitored daily for clinical signs and sampled for viral excretion over a 2-week period.

### 2.4. Measurement of NDV- and AIV-Specific Antibody Responses

NDV-specific antibodies were evaluated with (i) hemagglutination inhibition (HI) tests using La Sota NDV antigen [4], (ii) commercial IDVet ND-ELISAs (ID Screen^®^ Newcastle disease indirect (NDVS), IDVet) [4], and (iii) NDV-specific IgG in-house ELISAs [12]. AIV-specific antibody responses were evaluated in (i) HI tests using influenza hemagglutinin (HA) antigens of the challenge virus (*A/Chicken/Egypt/1709-6/2008*) (named challenge HA) and of the H5 insert in the rHVT-H5(AI) (*A/Swan/Hungary/4571/2006*) [10] (named vaccine HA), (ii) commercial IDVet H5-ELISA (ID kit Screen^®^ Influenza H5 Antibody Competition, IDVet) [13], and (iii) AIV-specific IgG in-house ELISAs [10].

### 2.5. Measurement of Viral Shedding after Challenge

Quantification of vNDV and H5-HPAIV challenge strains from oro-nasal/tracheal and cloacal swabs was conducted by quantitative real-time reverse transcription-polymerase chain reaction (QRRT-PCR) targeting NDV and AIV matrix (M) genes, respectively, as previously described [4,11]. NDV and AIV shedding results are expressed as log_10_ titer equivalent unit value (log_10_ EID_50_/0.2 mL) and number of M-AIV gene copy number per milliliter swab samples, respectively. The quality of the sample and RNA extraction procedure were validated using avian β-actin, as described previously [14].

### 2.6. Statistical Analysis

Data analysis was performed by using Minitab 13 (Minitab Ltd., Coventry, UK) and STATA 10 (Stata Corp LP, College Station, TX, USA) software. Differences between vaccination schedules or between MDA(–) and MDA(+) groups were considered significant at *p* < 0.05. In experiment I, antibody levels and viral excretion were compared at specific times between vaccination schedules by one-way analysis of variance (ANOVA) and Kruskal–Wallis tests with 95% confidence intervals (CIs), as described previously [15]. In experiment II, antibody levels and viral excretion were compared at specific times between (i) vaccination schedules and (ii) MDA(–)/MDA(+) groups with two-way ANOVA and multiple range tests with 95% CIs. In both experiments, qualitative criteria of “protection” (observed morbidity/mortality or not) and “positive QRRT-PCR reaction” (viral excretion or not) were analyzed by Fisher’s exact test with the Bonferroni method to adjust risk α [15] for comparison within (i) the vaccination schedules and (ii) the MDA groups. Finally, seroconversion of the vaccinated groups was reported to be significant if antibody levels were above the threshold of positivity and significantly higher compared with the unvaccinated group (*p* < 0.05).

## 3. Results

### 3.1. Clinical Protection

Levels of clinical protection obtained during consecutive vNDV and H5-HPAIV challenges in trials I and II are summarized in Table 1 and Table 2, respectively. At each challenge date, all of the chickens in the control group died, thereby validating both vNDV and H5-HPAIV challenges in both SPF WL and LBL layer birds.

#### 3.1.1. ND Clinical Protection

In SPF WL experiments, no difference in ND protection between ND and AI/ND vaccination schedules was observed, independent of the age at vNDV challenge (Table 1). Indeed, following vNDV challenges at 3 weeks and 8 weeks of age, clinical protection of 90% and 100% were recorded, respectively, independent of ND or AI/ND vaccination.

Among MDA(–) LBL chickens, clinical protection against vNDV reached 100% after ND vaccination, independent of the age at challenge (Table 2). Furthermore, AI/ND vaccination afforded 95% protection at 4 weeks and 100% protection at both 7 weeks and 10 weeks. In the MDA(+) group, 100% protection was observed after ND vaccination, independent of challenge timing. Following AI/ND vaccination, ND protection at 4, 7, and 10 weeks reached 85%, 95%, and 100%, respectively.

There was no significant difference in ND protection between the ND and AI/ND vaccination schedules in LBL chickens, independent of their MDA status and age at challenge. There was also no difference between the MDA(–) and MDA(+) groups, independent of the age at challenge and ND or AI/ND vaccination schedules.

#### 3.1.2. AI Clinical Protection

In SPF WL chicken experiments, clinical protection reached 100% at both 4 weeks and 8 weeks after AI vaccination, and reached 100% and 90% protection after AI/ND vaccination, respectively (Table 1).

In MDA(–) LBL chickens, clinical protection against H5-HPAIV reached 90% at 4 weeks and 100% at both 7 weeks and 10 weeks after AI vaccination (Table 2). After AI/ND vaccination, clinical protection at 4, 7, and 10 weeks was 70%, 90%, and 100%, respectively.

AI protection in MDA(+) LBL chickens decreased to 0% and 20% when a challenge with H5-HPAIV was conducted 4 weeks after AI and AI/ND vaccinations, respectively. At 7 weeks, AI protection reached 100% and 80%, respectively, and 80% and 90%, at 10 weeks.

There was no significant difference in AI protection between the MDA(–) and MDA(+) LBL groups after AI/ND vaccination, independent of their age of challenge. In contrast, significantly lower (*p* < 0.05) AI protection was reported in the MDA(+) group compared with the MDA(–) group at 4 weeks after AI vaccination, yet not at the older ages of challenge.

### 3.2. Viral Shedding after Challenge

#### 3.2.1. NDV Shedding

Independent of age at the vNDV challenge, both the ND and AI/ND vaccination schedules significantly reduced (*p* < 0.05) vNDV shedding at 3 days post-infection (dpi) by oro-nasal and cloacal routes in SPF WL chickens compared to the unvaccinated group (Table 3). In contrast, no difference was observed between the two vaccination schedules regarding oro-nasal or cloacal excretion, except at 3 dpi when challenged at 8 weeks, when the ND group shed significantly less (*p* < 0.05) via the oro-nasal route than the AI/ND group.

In MDA(–) LBL chickens, both ND and AI/ND vaccination schedules significantly reduced (*p* < 0.05) vNDV shedding via oro-nasal and cloacal routes at 3 dpi and 7 dpi, respectively, compared to the unvaccinated group, irrespective of age at vNDV challenge (Table 4). After a challenge at 7 weeks of age, ND vaccination reduced viral shedding more significantly (*p* < 0.05) via the oro-nasal route compared to AI/ND vaccination, regardless of the swabbing day. At the same age of challenge, significantly lower (*p* < 0.05) shedding was also reported via the cloacal route in the ND group at 3 dpi. In contrast, no difference in oro-nasal and cloacal excretion was observed between the vaccination schedules when challenges were performed at 4 weeks and 10 weeks of age.

In MDA(+) LBL chickens, both ND and AI/ND vaccination schedules significantly reduced (*p* < 0.05) vNDV shedding via both oro-nasal and cloacal routes at 3 dpi and 7 dpi compared to the unvaccinated group when challenged with vNDV at 7 weeks and 10 weeks (Table 4). At 4 weeks, oro-nasal excretion was significantly reduced (*p* < 0.05) by both ND and AI/ND vaccinations, independent of the swabbing day. Both vaccination schedules led to a significant reduction (*p* < 0.05) in cloacal shedding at 3 dpi compared to the unvaccinated group, while the ND vaccination group additionally exhibited significantly reduced (*p* < 0.05) cloacal shedding at 7 dpi. Another difference between the vaccination schedules was reported following the challenge at 7 weeks, with a stronger reduction (*p* < 0.05) in oro-nasal and cloacal excretions observed at 3 dpi and 7 dpi, respectively, in the ND group compared to the AI/ND group. In contrast, no difference in oro-nasal or cloacal shedding was observed between the two vaccination schedules after challenges at 4 weeks and 10 weeks. There was also no difference in shedding reported between the MDA(–) and MDA(+) groups, independent of the age at challenge or vaccination schedule.

#### 3.2.2. AIV Shedding

Independent of the age at H5-HPAIV challenge, both the AI and AI/ND vaccination schedules significantly reduced (*p* < 0.05) viral shedding via tracheal and cloacal routes at 2 dpi in SPF WL chickens compared to the unvaccinated group (Table 5). In contrast, no difference was observed between the two vaccination schedules regarding tracheal and cloacal shedding, except at 4 dpi when challenged at 8 weeks. At this time point, tracheal excretion was significantly lower (*p* < 0.05) in the AI group compared to the AI/ND group.

In MDA(–) LBL birds following H5-HPAIV challenge at 7 weeks and 10 weeks of age, both AI and AI/ND vaccination schedules significantly reduced (*p* < 0.05) shedding via tracheal and cloacal routes at 2 dpi compared to the unvaccinated group (Table 6 and Table 7). Furthermore, no difference between the vaccination schedules was reported at these ages of challenge. However, when challenged at 4 weeks, cloacal excretion at 2 dpi was significantly reduced (*p* < 0.05) in both the AI and AI/ND groups compared to the unvaccinated group (Table 8), while tracheal excretion was only significantly reduced (*p* < 0.05) with AI vaccination. No difference in shedding was observed between the two vaccination schedules, except at 2 dpi when tracheal excretion was significantly reduced (*p* < 0.05) in the AI group compared with the AI/ND group.

In MDA(+) LBL chickens, both AI and AI/ND vaccination schedules significantly reduced (*p* < 0.05) shedding via both tracheal and cloacal routes at 2 dpi compared with the unvaccinated group following H5-HPAIV challenges at 7 weeks and 10 weeks (Table 7 and Table 8). Furthermore, no difference between the vaccination schedules was reported at these ages of challenge. However, at 4 weeks, only the AI vaccination schedule led to a significant reduction (*p* < 0.05) in both tracheal and cloacal excretions at 4 dpi, and tracheal excretion alone at 2 dpi, compared to the unvaccinated group (Table 6). There was no difference in shedding between the two vaccination schedules, except at 2 dpi and 4 dpi when tracheal excretion was significantly reduced (*p* < 0.05) in the AI group compared with the AI/ND group.

When unvaccinated chickens were challenged at 4 weeks (Table 6) and 7 weeks (Table 7) of age with H5-HPAIV, both tracheal and cloacal shedding at 2 dpi were significantly reduced (*p* < 0.05) in the MDA(+) group compared to the MDA(–) group. For the AI/ND vaccination group which was challenged at 4 weeks (Table 6) and 10 weeks (Table 8), significantly (*p* < 0.05) increased tracheal and cloacal excretions were observed in MDA(+) chickens at 4 dpi compared to their MDA(–) counterparts. However, no difference between MDA(+) and MDA(–) chickens in the AI/ND vaccination group was reported at the 7 week challenge (Table 7). Similarly, no difference in shedding was observed in the AI vaccination group for any age at challenge.

### 3.3. Antibody Response

#### 3.3.1. NDV-Specific Antibody Response

**Figure 1:**
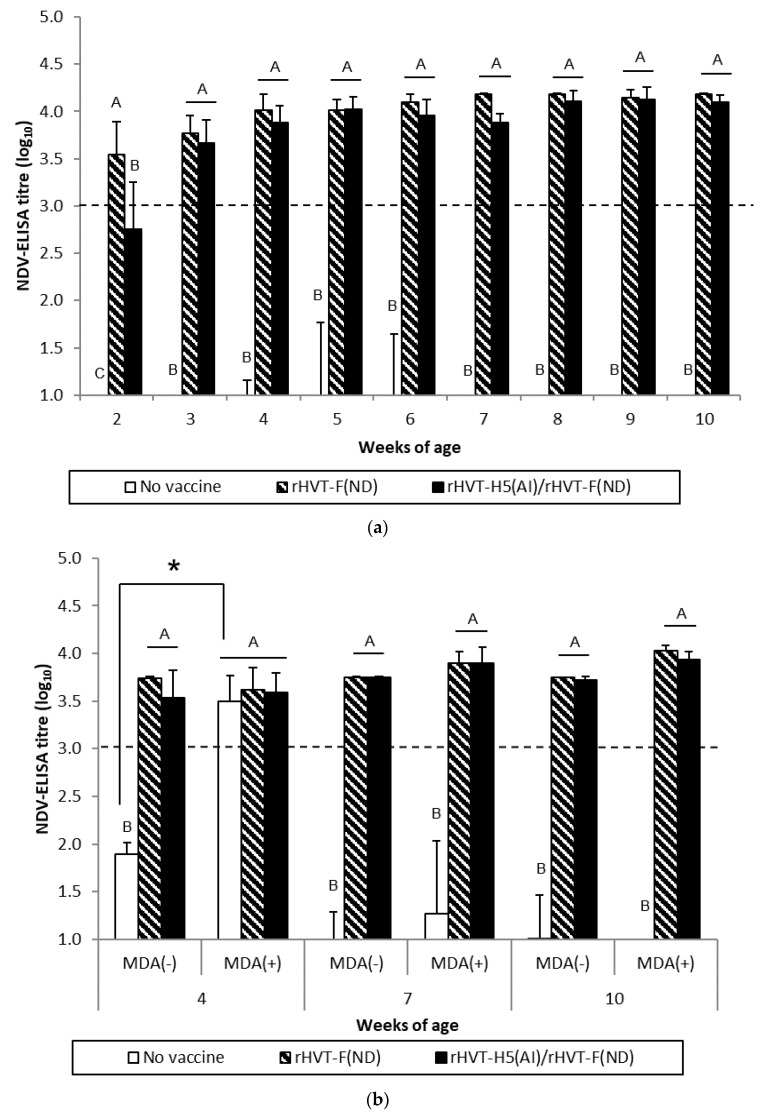
NDV-specific antibody responses measured in ND-ELISAs for WL SPF chickens (**a**) and LBL layer chickens (**b**) vaccinated at day-old (experiments I and II, respectively) with rHVT-F(ND) or both rHVT-H5(AI)/rHVT-F(ND) vaccines. Data represent mean ± standard deviation of antibody titers (log_10_) determined by ELISA at specified ages (n = 5). ND-ELISAs were performed according to the manufacturer’s recommendations. Titers ≥ 3.0 log_10_ were considered positive (this threshold of positivity is indicated by the dotted line). In figure (**a**), mean ± standard deviation at time points with no common uppercase letters (A, B, C) indicates a significant difference between vaccination schedules (*p* < 0.05). In figure (**b**), mean ± standard deviation at time points with no common uppercase letters (A, B) indicates a significant difference between vaccination schedules within the MDA group (*p* < 0.05), while asterisk superscript symbol (*) indicates a significant difference between MDA(–) and MDA(+) groups within a given vaccination schedule (*p* < 0.05).

**Figure 2:**
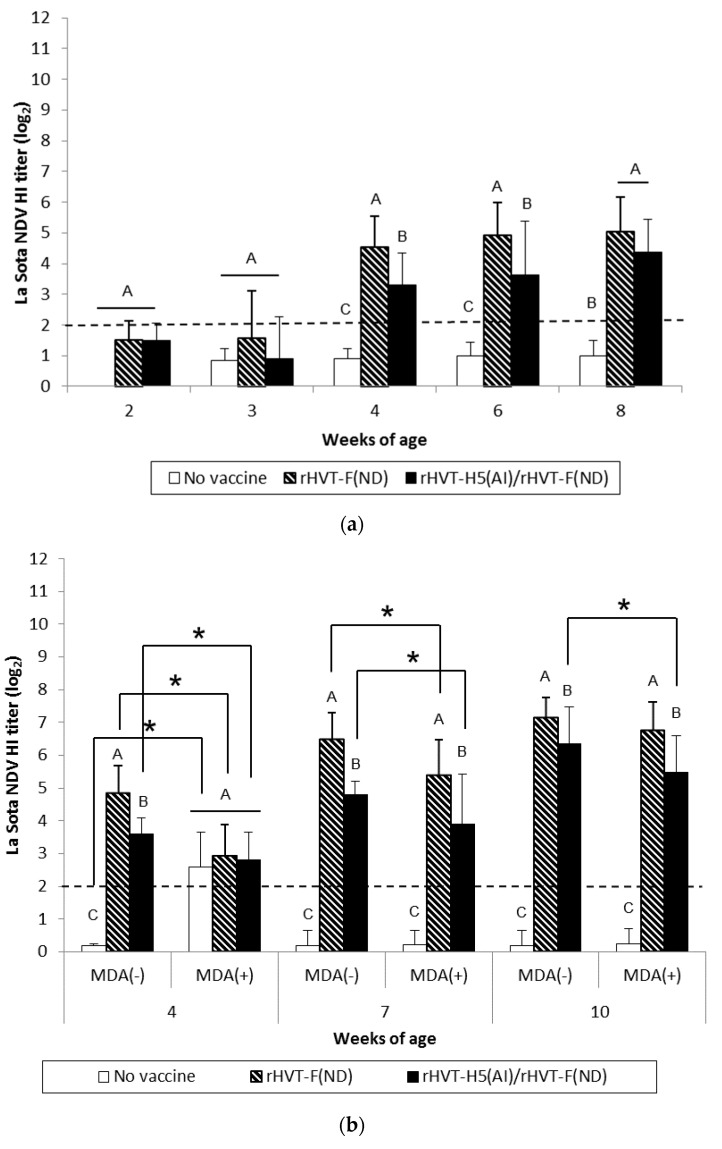
La Sota NDV-HI titers in serum of WL SPF chickens (**a**) and LBL layer chickens (**b**) vaccinated at day-old (experiments I and II, respectively) with rHVT-F(ND) or both rHVT-H5(AI)/rHVT-F(ND) vaccines. Data represent mean ± standard deviation (n = 10–20) of HI titers (log_2_), which correspond to the last dilution exhibiting inhibition of 4 hemagglutination units of the La Sota NDV antigen. The HI geometric mean titers are expressed as reciprocal log_2_. Titers ≥ 2 log_2_ were considered positive (this threshold of positivity is indicated by the dotted line). In figure (**a**), mean ± standard deviation at time points with no common uppercase letters (A, B, C) indicates a significant difference between vaccination schedules (*p* < 0.05). In figure (**b**), mean ± standard deviation at time points with no common uppercase letters (A, B, C) indicate a significant difference between vaccination schedules within the MDA group (*p* < 0.05), while asterisk superscript symbol (*) indicates a significant difference between MDA(–) and MDA(+) groups within a given vaccination schedule (*p* < 0.05).

WL SPF chickens in the ND and AI/ND groups exhibited significant (*p* < 0.05) NDV seroconversion from 2 weeks and 3 weeks of age, respectively, according to ND-ELISA results (Figure 1a). Seroconversion was subsequently detected at 4 weeks of age in HI tests. The HI titers of both the ND and AI/ND groups were significantly higher (*p* < 0.05) compared to the unvaccinated group (Figure 2a). Furthermore, ND vaccination induced significantly higher (*p* < 0.05) NDV-specific antibody response than AI/ND vaccination as observed (i) at 2 weeks by ND-ELISA and (ii) at 4 weeks and 6 weeks in HI tests.

ND vaccination of SPF WL chickens induced a NDV-specific antibody response detectable from two weeks of age onwards with commercial ND-ELISAs. This antibody response was delayed by one week in ND/AI group. This more gradual antibody response was corroborated in NDV-specific IgG in-house ELISAs (Figure S1), yet not in HI tests. Rather, the latter detected seroconversion only from 4 weeks of age, independent of vaccination schedule. Furthermore, HI titer remained more elevated at 4 weeks and 6 weeks in ND group in comparison to ND/AI group.

The NDV-specific antibody response was significantly higher (*p* < 0.05) in MDA(–) LBL layer chickens from 4 weeks of age in both the ND and AI/ND groups compared to the unvaccinated group, independent of the type of serological test performed (Figure 1b and Figure 2b). HI titers measured after ND vaccination were significantly higher (*p* < 0.05) than the titers obtained after AI/ND vaccination, regardless of age (Figure 2b), while no differences between vaccination schedules were detected by ND-ELISA (Figure 1b).

In MDA(+) LBL layer chickens, significant seroconversion (*p* < 0.05) was delayed from 7 weeks, independent of vaccination schedule and serological test. Furthermore, ND vaccination induced a significantly higher (*p* < 0.05) NDV-specific antibody response than AI/ND vaccination at each time point assayed by HI test, while not by ND-ELISA.

NDV-specific antibody immune responses measured by HI tests were significantly reduced (*p* < 0.05) in MDA(+) chickens compared to MDA(–) chickens at 4 weeks and 7 weeks of age, independent of vaccination schedule. In addition, only the HI titer induced by AI/ND vaccination remained significantly lower (*p* < 0.05) at 10 weeks in the MDA(+) group compared to the MDA(–) group. Furthermore, no differences between the MDA(–) and MDA(+) groups were detected in ND-ELISAs at any of the time points assayed.

#### 3.3.2. AIV-Specific Antibody Response

**Figure 3:**
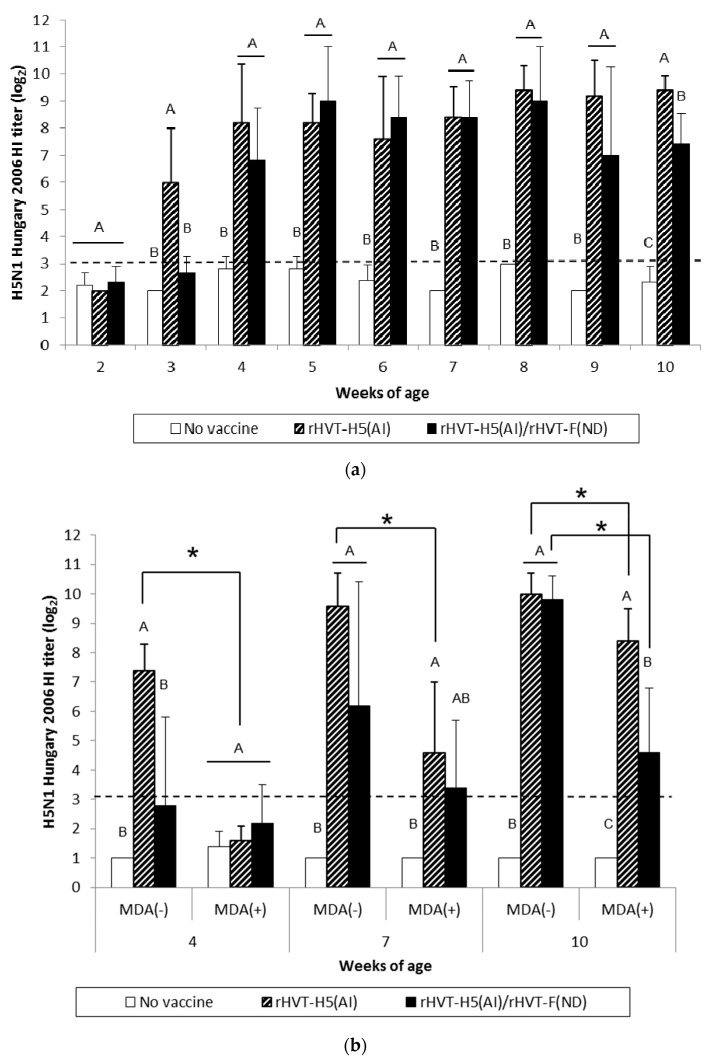
H5N1 Hungary 2006 HI titers in serum of WL SPF chickens (**a**) and LBL layer chickens (**b**) vaccinated at day-old (experiments I and II, respectively) with rHVT-H5(AI) or both rHVT-H5(AI)/rHVT-F(ND) vaccines. Data represent mean ± standard deviation (n = 5) of HI titers (log_2_), which correspond to the last dilution exhibiting inhibition of 4 hemagglutination units of *A/Swan/Hungary/4571/2006* AIV HA antigens. The HI geometric mean titers are expressed as reciprocal log_2_. Titers > 3 log_2_ were considered positive (this threshold of positivity is indicated by the dotted line). In figure (**a**), mean ± standard deviation at time points with no common uppercase letters (A, B, C) indicates a significant difference between vaccination schedules (*p* < 0.05). In figure (**b**), mean ± standard deviation at time points with no common uppercase letters (A, B, C) indicate a significant difference between vaccination schedules within the MDA group (*p* < 0.05), while asterisk superscript symbol (*) indicates a significant difference between MDA(–) and MDA(+) groups within a given vaccination schedule (*p* < 0.05).

**Figure 4:**
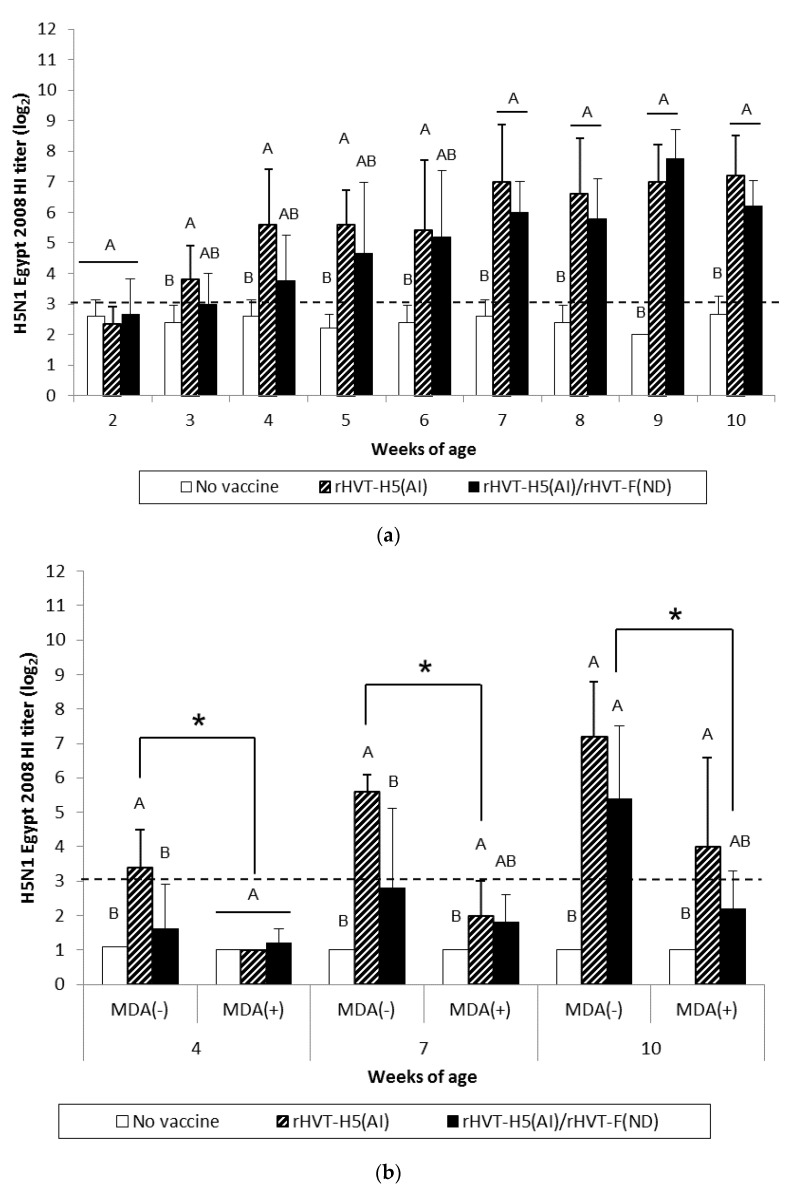
H5N1 Egypt 2008 HI titers in serum of WL SPF chickens (**a**) and LBL layer chickens (**b**) vaccinated at day-old (experiments I and II, respectively) with rHVT-H5(AI) or both rHVT-H5(AI)/rHVT-F(ND) vaccines. Data represent mean ± standard deviation (n = 5) of HI titers (log_2_), which correspond to the last dilution exhibiting an inhibition of 4 hemagglutination units of *A/Chicken/Egypt/1709-6/2008* AIV HA antigens. HI geometric mean titers are expressed as reciprocal log_2_. Titers > 3 log_2_ were considered positive (this threshold of positivity is indicated by the dotted line). In figure (**a**), mean ± standard deviation at time points with no common uppercase letters (A, B) indicates a significant difference between vaccination schedules (*p* < 0.05). In figure (**b**), mean ± standard deviation at time points with no common uppercase letters (A, B) indicates a significant difference between vaccination schedules within the MDA group (*p* < 0.05), while asterisk superscript symbol (*) indicates a significant difference between MDA(–) and MDA(+) groups within a given vaccination schedule (*p* < 0.05).

**Figure 5:**
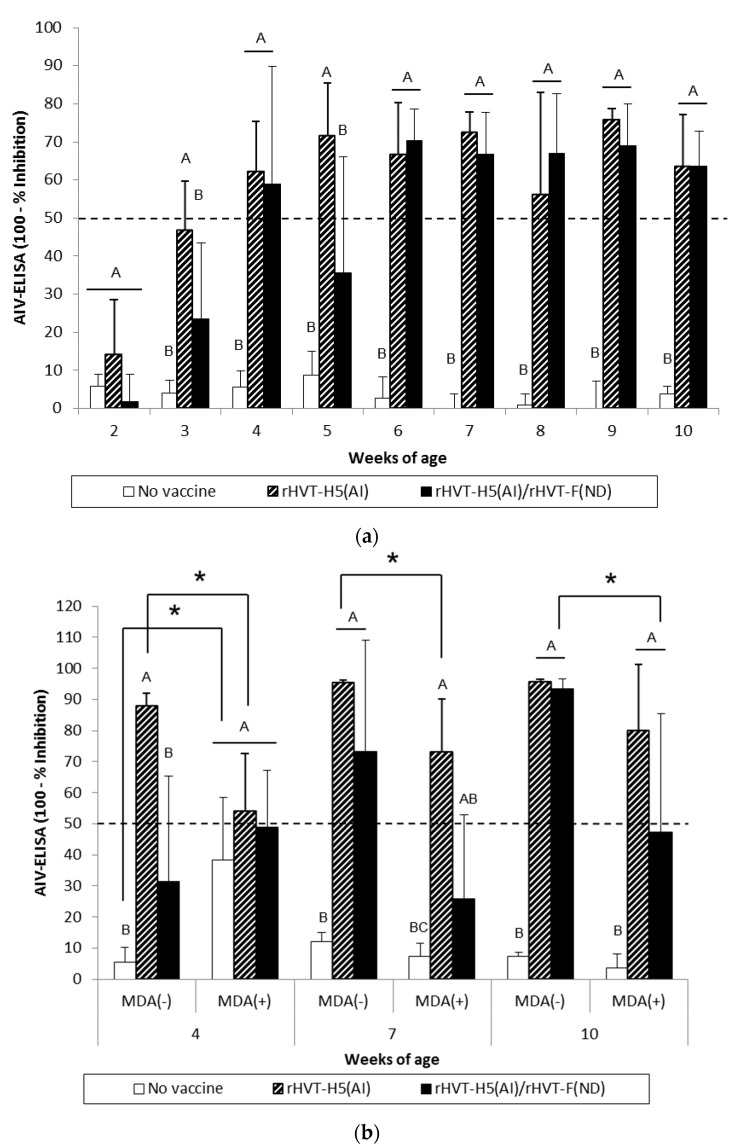
AIV-specific antibody responses measured in H5-ELISAs for WL SPF chickens (**a**) and LBL layer chickens (**b**) vaccinated at day-old (experiments I and II, respectively) with rHVT-F(ND) or both rHVT-H5(AI)/rHVT-F(ND) vaccines. Data represent mean ± standard deviation of antibody titers (log10) determined by ELISA at specified ages (n = 5). H5-ELISAs were performed according to the manufacturer’s recommendations. Inhibition ≥ 50% was considered positive (this threshold of positivity is indicated by the dotted line). In figure (**a**), mean ± standard deviation at time points with no common uppercase letters (A, B) indicates a significant difference between vaccination schedules (*p* < 0.05). In figure (**b**), mean ± standard deviation at time points with no common uppercase letters (A, B, C) indicate a significant difference between vaccination schedules within the MDA group (*p* < 0.05), while asterisk superscript symbol (*) indicates a significant difference between MDA(–) and MDA(+) groups within a given vaccination schedule (*p* < 0.05).

AI vaccination of WL SPF chickens resulted in significant seroconversion (*p* < 0.05) against vaccine HA antigen (H5N1 Hungary 2006) by 3 weeks of age compared to the unvaccinated group (Figure 3a). Furthermore, significant seroconversion (*p* < 0.05) was delayed until 4 weeks of age in the AI/ND vaccination group. When the challenge HA antigen (H5N1 Egypt 2008) was used, HI titers were significantly higher (*p* < 0.05) from 3 weeks of age in the AI-vaccinated group (Figure 4a). A significant increase (*p* < 0.05) in HI titers was not observed until 7 weeks of age in the AI/ND vaccination group compared to the unvaccinated group (Figure 4a). AI seroconversion (*p* < 0.05) was also detected starting at 4 weeks of age by H5-ELISA, independent of vaccination schedule (Figure 5a).

At certain time points, AI vaccination induced a significantly higher (*p* < 0.05) AIV-specific antibody response than AI/ND vaccination. These differences between AI and AI/ND vaccination were observed (i) at 3 weeks and 10 weeks in HI tests using vaccine HA antigen, and (ii) at 3 weeks and 5 weeks in H5-ELISA. There was no difference between AI and AI/ND vaccinations throughout the observation period according to HI tests conducted with challenge HA antigen.

After AI vaccination of SPF WL chickens, an AIV-specific antibody response was detected in HI tests from 3 weeks of age whenever the HA antigen was used, and from 4 weeks of age by both H5-ELISAs and AIV-specific IgG in-house ELISAs (Figure S2). Similar to the NDV-specific antibody response, the AIV-specific antibody response also rose more slowly in ND/AI group and was detected from 1 to 4 weeks later, depending on the serological test used.

MDA(–) LBL layer birds exhibited a significant seroconversion (*p* < 0.05) against vaccine HA antigen from 4 weeks and 7 weeks of age in the AI and AI/ND groups, respectively (Figure 3b). The same onset of AI seroconversion was reported by H5-ELISA (Figure 5b). Seroconversion (*p* < 0.05) against challenge HA antigen was also observed from 4 weeks in the AI group yet was delayed until 10 weeks in the AI/ND group (Figure 4b). Furthermore, AI vaccination induced a significantly higher (*p* < 0.05) AIV-specific antibody response than AI/ND vaccination at 4 weeks in HI tests using vaccine HA antigen and in H5-ELISA, and at 4 weeks and 7 weeks in HI tests using challenge HA antigen.

In MDA(+) LBL layer chickens, significant seroconversion (*p* < 0.05) induced by AI vaccination was detected from 7 weeks of age, independent of the serological test used (Figure 3b, Figure 4b, and Figure 5b). Seroconversion in the AI/ND group was delayed until 10 weeks of age and was only detected in HI tests using vaccine HA antigen and in H5-ELISAs. In contrast, no seroconversion against challenge HA antigen was observed at any of the time points tested. AI vaccination induced a significantly higher (*p* < 0.05) AIV-specific antibody response than AI/ND vaccination at 10 weeks only in HI tests using vaccine HA antigen.

Antibody response induced by AI vaccination was significantly reduced (*p* < 0.05) in the MDA(+) group compared with the MDA(–) group at 4 weeks and 7 weeks of age, independent of serological test. In addition, the HI titer of the MDA(+) group against vaccine HA antigen remained significantly lower (*p* < 0.05) at 10 weeks. Less seroconversion was observed in the MDA(+) group after AI/ND vaccination at certain time points compared to the MDA(–) group at 10 weeks in both HI tests (vaccine and challenge HA antigens) and H5-ELISAs.

## 4. Discussion

### 4.1. Comparable Clinical Protection and Reduction of Challenge Virus Shedding

Simultaneous vaccination with rHVT-F(ND) and rHVT-H5(AI) in a single shot at day-old afforded clinical protection in SPF WL and LBL layer chickens against both vNDV and H5-HPAIV challenges. Thus, the European Pharmacopeia requirements for single ND and AI vaccination are fulfilled [16]. A marked reduction in viral shedding was also reported by the combined rHVT-F(ND) and rHVT-H5(AI) vaccines, except only for tracheal excretion at 2 dpi following a challenge at 4 weeks of age with H5-HPAIV.

However, in our experiments, the H5-HPAIV protection rate observed at 4 weeks in rHVT-H5(AI) vaccinated MDA(+) LBL layer chickens was markedly lower than previously observed [10,17], and also reported, when combined with the rHVT-F(ND) vaccine. This may be due to differences in the vaccination programs used in breeders, and/or the impact of homologous/heterologous H5-strains used for breeders and offspring vaccination. Differences in genetic backgrounds and the immune responses between layer- and broiler-type chickens [18] may also contribute to differences in protection. For example, a host’s genetic background has been shown to modulate vaccine efficiency for MD [19], H5-HPAIV [20], and H9N2 LPAIV [21]. The cell-associated form of rHVT vaccines are known to spread from infected cells to uninfected cells via direct interactions, while enveloped virions may also bind to cellular receptors via gB proteins in combination with other HVT glycoproteins [1].

### 4.2. Delayed Antibody Response

Both ND- and AIV-specific antibody responses were delayed when SPF WL and LBL layer chickens were vaccinated with both rHVT-F(ND) and rHVT-H5(AI) vaccines in a single shot, compared to administration of the vaccines individually. These results demonstrate that administration of rHVT-F(ND) and rHVT-H5(AI) vaccines individually, or in combination, triggers other arms of the immune response than serological antibody response, such as cell-mediated immunity ([22,23] personal observation) and local antibody response [personal observation], to ensure a high level of clinical protection and reduced viral shedding following vNDV or H5-HPAIV challenge. While these results are consistent with previously reported results for single vaccinations of rHVT-F(ND) and rHVT-H5(AI) [4,11,23], the delayed onset in ND- and AIV-specific antibody responses after administration of both rHVT-F(ND) and rHVT-H5(AI) vaccines in single shot remains to be explained. Our hypothesis is that a mixture of rHVT vaccines would lead to competition and reduced take-up and replication of one rHVT vaccine. Although both rHVT-F(ND) and rHVT-H5(AI) vaccines are derived from the same parental strain of HVT, minor changes may be enough to cause a competitive disadvantage for antigen expression and antibody-eliciting capacity [24,25,26]. It is possible that an insufficient number of target cells are present at the inoculation site at day-old to take up both vaccines, leading to exclusion of one of the vectors from target cells and to less induction of an early immune response by this combined vaccination schedule. For this, a comparison of day-old chicken vaccinations with one dose each of rHVT-F(ND) and rHVT-H5(AI) in the neck at separate sites (double shot) versus a single shot could be performed. Detection of rHVT-F(ND) and rHVT-H5(AI) genome loads in feather follicles and organs by F- and H5- or HVT-specific real-time quantitative PCR methods [4,9,18] could further provide quantitative data regarding vaccine replication and distribution.

### 4.3. Interference from MDA

Our experiments conducted in MDA(–) and MDA(+) LBL layer chickens clearly highlight stronger interference of AIV-specific MDA than NDV-specific MDA with corresponding rHVT-H5(AI) or rHVT-F(ND) vaccinations, as well as related H5-HPAIV/vNDV infections. Interestingly, a significant reduction in excretion was afforded by AIV-specific MDA until 7 weeks of age when unvaccinated chickens were challenged with H5-HPAIV. In contrast, NDV-specific MDA does not appear to reduce viral shedding following infection with vNDV. This stronger protective effect of AIV-specific MDA against viral infection is consistent with previous observations [27] and may be explained by two hypotheses. First, AIV enters host cells via an endocytic route in a pH-dependent manner, while NDV enters host cells via direct cell plasma membrane fusion in a pH-independent manner, as previously reviewed [28,29]. Second, AIV uses a single glycoprotein, namely HA, to mediate host receptor binding and virus entry [30,31], while NDV requires two distinct glycoproteins, HN and F, respectively [32,33,34]. Regarding the latter, H5-AIV-specific antibodies would then mediate greater neutralizing activity and greater protection against viral infection than NDV-specific antibodies. In addition, the latter would reduce, yet not completely prevent, cell membrane fusion, and therefore, an induction of immune responses.

Cell-associated forms of rHVT-F(ND) and rHVT-H5(AI) vaccines have been shown to protect chickens, even in the presence of MDA, directed against both the HVT vector and the expressed F/H5 insert [4,10,17,22,35]. However, to date, no specific investigations of MDA directed against NDV and AIV have been reported. The experiments conducted in the present study with MDA(–) and MDA(+) LBL layer chickens clearly demonstrate the negative impact of specific passive immunity on the onset of antibody responses against NDV and AIV. Onset of protection against H5-HPAIV, yet not against vNDV challenge, was also delayed. Since both day-old MDA(–) and MDA(+) LBL layer birds are expected to have equal levels of MDA against HVT at the time of vaccination, these effects could certainly be attributed to MDA directed against F/H5 expressed proteins. The present findings suggest that NDV- and AIV-specific MDA interact differently with rHVT-F(ND) and rHVT-H5(AI) vaccines, and this results in differing consequences for efficacy. Both F and H5 protein genes are inserted in their native form in the rHVT-F(ND) and rHVT-H5(AI) vaccine constructs, respectively, with the exception of the cleavage site in the H5 gene, which was altered to represent a typical cleavage site sequence of an LP AIV strain. Therefore, it may be that F and H5 proteins are expressed at the surface of rHVT-F(ND) and rHVT-H5(AI) virions, respectively, as part of their respective parental enveloped viruses. Due to their bivalent character, these two rHVT vaccines could theoretically use both HVT and NDV/AIV pathways for host cell attachment and/or penetration. This would be promoted by F and H5 proteins via distinct mechanisms, respectively, as mentioned above for NDV and AIV. MDA directed against NDV and AIV could consequently (i) reduce the presentation of F and H5 antigens by fixing and hiding specific epitopes of the rHVT-F(ND) and rHVT-H5(AI) vaccines, and (ii) neutralize the vaccine by reducing its replication, thereby hampering specific active immune responses. The latter has been observed for other attenuated vaccines [36,37,38,39,40,41,42]. Considering that H5-AIV-specific MDA are more neutralizing than NDV-specific MDA [27], it is reasonable to conclude that the interference of the former with the induction of immune responses by rHVT-H5(AI) vaccination is stronger than the interference of the latter with the induction of immune responses by rHVT-F(ND) vaccination. Further immunophenotyping experiments could be performed with cells isolated from chickens vaccinated with rHVT-F(ND) and rHVT-H5(AI), where quantitative detection of target and infected cells would be performed and mechanism(s) of immune response induction in animals with and without MDA would be investigated.

## 5. Conclusions

Our findings indicate that rHVT-F(ND) and rHVT-H5(AI) vaccines can be administered simultaneously in a single shot to day-old chickens to protect them against clinical signs of both vNDV and H5-HPAIV infections. Marked reductions in viral shedding were also achieved. Thus, vaccine requirements of the European Pharmacopeia are met. However, this combination of vaccines may generate slightly lower levels of NDV- and AIV-specific antibodies. The present findings suggest that there are negative impacts of NDV- and AIV-specific MDA on the onset of specific antibody responses induced by day-old vaccinations with rHVT-F(ND) and rHVT-H5(AI), respectively, and on the onset of protection against H5-HPAIV but not against vNDV challenge. Thus, additional studies are needed to determine the mechanism mediating this MDA interference and its extent on cell-mediated immunity and local immune response. Finally, implementation of post-vaccination monitoring programs is needed to check for proper vaccine uptake and/or induced protection.

## Figures and Tables

**Table 1:** Clinical protection against challenges with vNDV and H5-HPAIV strains in SPF WL chickens vaccinated at day-old with rHVT-F(ND), rHVT-H5(AI), or both (experiment I).

Vaccine	ND Protection (%)		AI Protection (%)	
	3 Weeks	8 Weeks	4 Weeks	8 Weeks
No vaccine	0 ^B^	0 ^B^	0 ^B^	0 ^B^
rHVT-H5(AI)	N.D.	N.D.	100 ^A^	100 ^A^
rHVT-F(ND)	90 ^A^	100 ^A^	N.D.	N.D.
rHVT-H5(AI)/rHVT-F(ND)	90 ^A^	100 ^A^	100 ^A^	90 ^A^

Different uppercase superscript letters (A, B) indicate a significant difference (*p* < 0.05) between vaccination schedules within week of challenge (per column). N.D.: not determined.

**Table 2:** Clinical protection against challenges with vNDV and H5-HPAIV strains in LBL layer chickens vaccinated at day-old with rHVT-F(ND), rHVT-H5(AI), or both (experiment II).

Weeks	Vaccine	ND Protection (%)		AI Protection (%)	
		MDA(–)	MDA(+)	MDA(–)	MDA(+)
4	No vaccine	0 ^B^	0 ^B^	0 ^B^	0 ^A^
	rHVT-H5(AI)	N.D.	N.D.	90 ^A^	0 ^A^ *
	rHVT-F(ND)	100 ^A^	100 ^A^	N.D.	N.D.
	rHVT-H5(AI)/rHVT-F(ND)	95 ^A^	85 ^A^	70 ^A^	20 ^A^
7	No vaccine	0 ^B^	0 ^B^	0 ^B^	0 ^B^
	rHVT-H5(AI)	N.D.	N.D.	100 ^A^	100 ^A^
	rHVT-F(ND)	100 ^A^	100 ^A^	N.D.	N.D.
	rHVT-H5(AI)/rHVT-F(ND)	100 ^A^	95 ^A^	90 ^A^	80 ^A^
10	No vaccine	0 ^B^	0 ^B^	0 ^B^	0 ^B^
	rHVT-H5(AI)	N.D.	N.D.	100 ^A^	80 ^A^
	rHVT-F(ND)	100 ^A^	100 ^A^	N.D.	N.D.
	rHVT-H5(AI)/rHVT-F(ND)	100 ^A^	100 ^A^	100 ^A^	90 ^A^

Different uppercase superscript letters (A, B) indicate a significant difference (*p* < 0.05) between vaccination schedules within MDA groups (per column), while asterisk superscript symbol (*) indicates a significant difference (*p* < 0.05) between MDA(–) and MDA(+) groups within vaccination schedule (per line). N.D.: not determined.

**Table 3:** Oro-nasal and cloacal shedding of vNDV after challenges at 3 weeks and 8 weeks of age with vNDV to SPF WL chickens (experiment I) vaccinated at day-old with rHVT-F(ND), rHVT-H5(AI), or both vaccines.

Weeks	Vaccine	Days after Challenge ^a,b^			
		Oro-Nasal Shedding		Cloacal Shedding	
		3	7	3	7
3	No vaccine	6.88 ± 0.49 ^A^	N.D.	6.95 ± 0.41 ^A^	N.D.
		10/10	-	10/10	-
	rHVT-F(ND)	3.51 ± 0.68 ^B^	2.47 ± 1.11 ^A^	0.85 ± 1.73 ^B^	0.93 ± 1.56 ^A^
		10/20	16/18	5/20	5/18
	rHVT-H5(AI)/	4.79 ± 0.77 ^B^	3.59 ± 1.63 ^A^	0.58 ± 1.52 ^B^	3.03 ± 1.93 ^A^
	rHVT-F(ND)	20/20	20/20	3/20	16/20
8	No vaccine	5.74 ± 0.40 ^A^	N.D.	5.53 ± 0.95 ^A^	N.D.
		10/10	-	10/10	-
	rHVT-F(ND)	1.74 ± 1.16 ^C^	1.84 ± 2.04 ^A^	0.00 ± 0.00 ^B^	0.18 ± 0.56 ^A^
		8/10	5/10	0/10	1/10
	rHVT-H5(AI)/	3.54 ± 0.47 ^B^	2.46 ± 1.11 ^A^	0.00 ± 0.00 ^B^	0.00 ± 0.00 ^A^
	rHVT-F(ND)	10/10	9/10	0/10	0/10

^a^ Data presented as mean ± standard deviation of titer equivalent unit value (EID_50_) in milliliters of swabs (log_10_) as determined by QRRT-PCR of 1 mL swabs taken at specific times after challenge. Different uppercase superscript letters (A, B, C) indicate a significant difference (*p* < 0.05) between vaccination schedules at specific times after challenge (per column). ^b^ Data presented as frequency (number of virus-positive chickens/total tested chickens). Total numbers of chickens tested were reduced with time because of specific mortality. N.D.: not determined because of specific mortality due to challenge.

**Table 4:** Oro-nasal and cloacal shedding of vNDV after challenge at 4, 7, and 10 weeks of age with the vNDV of LBL layer chickens (experiment II) vaccinated at day-old with the rHVT-F(ND), the rHVT-H5(AI) or both vaccines.

Weeks	Dpi	Vaccine	Oro-Nasal Shedding ^a,b^		Cloacal Shedding ^a,b^	
			MDA(–)	MDA(+)	MDA(–)	MDA(+)
4	3	No vaccine	4.97 ± 0.57 ^A^	4.38 ± 0.67 ^A^	4.18 ± 1.07 ^A^	2.07 ± 1.19 ^A^
			10/10	10/10	10/10	6/10
		rHVT-F(ND)	2.70 ± 1.31 ^B^	2.61 ± 1.51 ^B^	0.46 ± 0.81 ^B^	0.22 ± 0.70 ^B^
			8/10	8/10	1/10	1/10
		rHVT-H5(AI)/	2.95 ± 1.22 ^B^	2.78 ± 1.76 ^B^	0.47 ± 0.71 ^B^	0.96 ± 1.42 ^B^
		rHVT-F(ND)	9/10	8/10	3/10	4/10
	7	No vaccine	5.56 ± 0.39 ^A^	6.43 ± 0.35 ^A^	6.64 ± 0.28 ^A^	5.98 ± 0.84 ^A^
			10/10	10/10	10/10	10/10
		rHVT-F(ND)	1.70 ± 1.16 ^B^	2.73 ± 0.65 ^B^	0.21 ± 0.54 ^B^	0.42 ± 1.34 ^B^
			6/10	10/10	1/10	1/10
		rHVT-H5(AI)/	2.18 ± 1.27 ^B^	2.72 ± 1.67 ^B^	0.19 ± 0.33 ^B^	1.61 ± 2.01 ^AB^
		rHVT-F(ND)	7/10	9/10	1/10	5/10
7	3	No vaccine	4.90 ± 0.34 ^A^	5.23 ± 0.63 ^A^	4.27 ± 0.39 ^A^	4.24 ± 1.32 ^A^
			5/5	10/10	5/5	10/10
		rHVT-F(ND)	0.99 ± 1.31 ^C^	1.42 ± 1.41 ^C^	0.00 ± 0.00 ^B^	0.00 ± 0.00 ^B^
			4/10	5/10	0/10	0/10
		rHVT-H5(AI)/	2.07 ± 1.37 ^B^	2.68 ± 1.58 ^B^	0.04 ± 0.14 ^B^	0.00 ± 0.00 ^B^
		rHVT-F(ND)	7/10	7/10	0/10	0/10
	7	No vaccine	7.32 ± 0.23 ^A^	7.32 ± 0.35 ^A^	7.13 ± 0.35 ^A^	6.93 ± 0.46 ^A^
			5/5	10/10	5/5	10/10
		rHVT-F(ND)	1.70 ± 0.91 ^C^	2.95 ± 1.52 ^B^	0.54 ± 0.59 ^C^	0.00 ± 0.00 ^C^
			8/10	9/10	2/10	0/10
		rHVT-H5(AI)/	3.65 ± 1.12 ^B^	3.45 ± 0.88 ^B^	1.56 ± 1.34 ^B^	1.51 ± 1.10 ^B^
		rHVT-F(ND)	10/10	10/10	6/10	6/10
10	3	No vaccine	4.86 ± 0.55 ^A^	4.82 ± 0.54 ^A^	4.10 ± 0.83 ^A^	3.89 ± 0.06 ^A^
			5/5	5/5	5/5	5/5
		rHVT-F(ND)	0.46 ± 0.81 ^B^	1.04 ± 1.37 ^B^	0.04 ± 0.13 ^B^	0.00 ± 0.00 ^B^
			2/10	4/10	0/10	0/10
		rHVT-H5(AI)/	0.00 ± 0.00 ^B^	1.95 ± 1.34 ^B^	0.00 ± 0.00 ^B^	0.00 ± 0.00 ^B^
		rHVT-F(ND)	0/10	7/10	0/10	0/10
	7	No vaccine	6.60 ± 0.53 ^A^	6.63 ± 0.27 ^A^	6.85 ± 0.44 ^A^	6.59 ± 0.33 ^A^
			5/5	5/5	5/5	5/5
		rHVT-F(ND)	2.35 ± 0.69 ^B^	1.96 ± 1.54 ^B^	0.00 ± 0.00 ^B^	0.00 ± 0.00 ^B^
			8/10	7/10	0/10	0/10
		rHVT-H5(AI)/	1.96 ± 1.50 ^B^	1.62 ± 1.56 ^B^	0.00 ± 0.00 ^B^	0.07 ± 0.23 ^B^
		rHVT-F(ND)	7/10	5/10	0/10	0/10

^a^ Data presented as mean ± standard deviation of titer equivalent unit value (EID_50_) in milliliters of swabs (log_10_) as determined by QRRT-PCR of 1 mL swabs taken at specific times after challenge. Different uppercase superscript letters (A, B, C) indicate a significant difference (*p* < 0.05) between vaccination schedules within MDA groups (per column). ^b^ Data presented as frequency (number of virus-positive chickens/total tested chickens). Total numbers of chickens tested were reduced with time because of specific mortality. N.D.: not determined because of specific mortality due to challenge.

**Table 5:** Tracheal and cloacal shedding of HPAIV after challenges at 4 and 8 weeks of age with H5-HPAIV administered to SPF WL chickens (experiment I) vaccinated at day-old with rHVT-F(ND), rHVT-H5(AI), or both vaccines.

Weeks	Vaccine	Tracheal Shedding According to Days after Challenge ^a,b^			
		2	4	7	10
4	No vaccine	7.48 ± 0.68 ^A^	N.D.	N.D.	N.D.
		3/3	-	-	-
	rHVT-H5(AI)	3.83 ± 2.80 ^B^	4.05 ± 2.94 ^A^	1.52 ± 2.56 ^A^	1.17 ± 1.92 ^A^
		7/10	7/10	3/10	3/10
	rHVT-H5(AI)/	3.20 ± 2.7 ^B^	3.81 ± 2.78 ^A^	1.25 ± 2.05 ^A^	0.00 ± 0.00 ^A^
	rHVT-F(ND)	7/10	7/10	7/10	7/10
8	No vaccine	8.14 ± 0.50 ^A^	N.D.	N.D.	N.D.
		9/9	-	-	-
	rHVT-H5(AI)	1.84 ± 2.40 ^B^	0.92 ± 1.95 ^B^	0.00 ± 0.00 ^A^	0.35 ± 1.11 ^A^
		4/10	2/10	0/10	1/10
	rHVT-H5(AI)/	3.71 ± 2.59 ^B^	5.20 ± 2.04 ^A^	0.93 ± 1.95 ^A^	0.50 ± 1.59 ^A^
	rHVT-F(ND)	7/10	9/10	2/10	1/10
**Weeks**	**Vaccine**	**Cloacal Shedding According to Days after Challenge ^a,b^**			
		**2**	**4**	**7**	**10**
4	No vaccine	5.76 ± 0.46 ^A^	N.D.	N.D.	N.D.
		3/3	-	-	-
	rHVT-H5(AI)	0.00 ± 0.00 ^B^	1.02 ± 2.20 ^A^	1.11 ± 2.35 ^A^	0.85 ± 1.89 ^A^
		0/10	2/10	2/10	2/10
	rHVT-H5(AI)/	0.00 ± 0.00 ^B^	0.00 ± 0.00 ^A^	0.00 ± 0.00 ^A^	0.00 ± 0.00 ^A^
	rHVT-F(ND)	0/10	0/10	0/10	0/10
8	No vaccine	6.09 ± 0.65 ^A^	N.D.	N.D.	N.D.
		9/9	-	-	-
	rHVT-H5(AI)	0.00 ± 0.00 ^B^	0.00 ± 0.00 ^A^	0.00 ± 0.00 ^A^	0.30 ± 0.94 ^A^
		0/10	0/10	0/10	1/10
	rHVT-H5(AI)/	0.00 ± 0.00 ^B^	0.51 ± 1.62 ^A^	0.00 ± 0.00 ^A^	0.00 ± 0.00 ^A^
	rHVT-F(ND)	0/10	1/10	0/10	0/10

^a^ Data presented as mean ± standard deviation of titer equivalent unit value (EID_50_) in milliliters of swabs (log_10_) as determined by QRRT-PCR of 1 mL swabs taken at specific times after challenge. Different uppercase superscript letters (A, B) indicate a significant difference (*p* < 0.05) between vaccination schedules at specific times after challenge (per column). ^b^ Data presented as frequency (number of virus-positive chickens/total tested chickens). Total numbers of chickens tested were reduced with time because of specific mortality. N.D.: not determined because of specific mortality due to challenge.

**Table 6:** Tracheal and cloacal shedding of H5-HPAIV after challenge at 7 weeks of age with H5-HPAIV to LBL layer chickens (experiment II) vaccinated at day-old with rHVT-F(ND), rHVT-H5(AI), or both vaccines.

Dpi	Vaccine	7 Weeks			
		Tracheal Shedding ^a,b^		Cloacal Shedding ^a,b^	
		MDA(–)	MDA(+)	MDA(–)	MDA(+)
2	No vaccine	8.18 ± 0.46 ^A^ *	6.99 ± 1.07 ^A^	6.40 ± 0.75 ^A^ *	5.47 ± 0.54 ^A^
		10/10	10/10	10/10	10/10
	rHVT-H5(AI)	3.55 ± 2.00 ^B^	3.63 ± 1.99 ^B^	0.47 ± 0.90 ^B^	0.26 ± 0.83 ^B^
		8/10	9/10	3/10	1/10
	rHVT-H5(AI)/	2.83 ± 2.70 ^B^	3.05 ± 3.11 ^B^	0.08 ± 0.27 ^B^	1.42 ± 2.43 ^B^
	rHVT-F(ND)	6/10	6/10	1/10	3/10
4	No vaccine	N.D.	N.D.	N.D.	N.D.
		-	-	-	-
	rHVT-H5(AI)	0.74 ± 1.59 ^A^	0.00 ± 0.00 ^A^	0.00 ± 0.00 ^A^	0.00 ± 0.00 ^A^
		2/10	0/10	0/10	0/10
	rHVT-H5(AI)/	1.67 ± 1.69 ^A^	0.36 ± 1.14 ^A^	0.00 ± 0.00 ^A^	0.00 ± 0.00 ^A^
	rHVT-F(ND)	6/10	1/10	0/10	0/10
7	No vaccine	N.D.	N.D.	N.D.	N.D.
		-	-	-	-
	rHVT-H5(AI)	0.00 ± 0.00 ^A^	1.10 ± 1.80 ^A^	0.00 ± 0.00 ^A^	0.00 ± 0.00 ^A^
		0/10	3/10	0/10	0/10
	rHVT-H5(AI)/	0.45 ± 1.34 ^A^	0.92 ± 1.94 ^A^	0.00 ± 0.00 ^A^	0.00 ± 0.00 ^A^
	rHVT-F(ND)	1/9	2/10	0/9	0/10
10	No vaccine	N.D.	N.D.	N.D.	N.D.
		-	-	-	-
	rHVT-H5(AI)	0.28 ± 0.87 ^A^	1.08 ± 1.80 ^A^	0.00 ± 0.00 ^A^	0.00 ± 0.00 ^A^
		1/10	3/10	0/10	0/10
	rHVT-H5(AI)/	0.49 ± 1.46 ^A^	1.00 ± 1.61 ^A^	0.00 ± 0.00 ^A^	0.00 ± 0.00 ^A^
	rHVT-F(ND)	1/9	3/10	0/9	0/10

^a^ Data presented as mean ± standard deviation of titer equivalent unit value (EID_50_) in milliliters of swabs (log_10_) as determined by QRRT-PCR of 1 mL swabs taken at specific times after challenge. Different uppercase superscript letters (A, B) indicate a significant difference (*p* < 0.05) between vaccination schedules within MDA groups (per column). The asterisk superscript symbol (*) indicates a significant difference (*p* < 0.05) between MDA(–) and MDA(+) groups within vaccination schedule (per line). ^b^ Data presented as frequency (number of virus-positive chickens/total tested chickens). Total numbers of chickens tested were reduced with time because of specific mortality. N.D.: not determined because of specific mortality due to challenge.

**Table 7:** Tracheal and cloacal shedding of H5-HPAIV after challenge and at 10 weeks of age with H5-HPAIV to LBL layer chickens (experiment II) vaccinated at day-old with rHVT-F(ND), rHVT-H5(AI), or both vaccines.

Dpi	Vaccine	10 Weeks			
		Tracheal Shedding ^a,b^		Cloacal Shedding ^a,b^	
		MDA(–)	MDA(+)	MDA(–)	MDA(+)
2	No vaccine	7.50 ± 0.29 ^A^	6.57 ± 2.56 ^A^	5.59 ± 0.51 ^A^	5.15 ± 1.98 ^A^
		10/10	8/9	10/10	8/9
	rHVT-H5(AI)	4.01± 1.73 ^B^	3.26 ± 2.03 ^B^	0.56 ± 1.18 ^B^	0.00 ± 0.00 ^B^
		9/10	8/10	2/10	0/10
	rHVT-H5(AI)/	2.81 ± 2.50 ^B^	2.14 ± 2.92 ^B^	0.00 ± 0.00 ^B^	0.56 ± 1.77 ^B^
	rHVT-F(ND)	6/10	4/10	0/10	1/10
4	No vaccine	N.D.	7.27 ^A^	N.D.	6.40 ± 0.73 ^A^
		--	1/1	-	1/10
	rHVT-H5(AI)	2.27 ± 1.72 ^A^	2.91 ± 1.77 ^A^	0.23 ± 0.00 ^A^	0.00 ± 0.00 ^C^
		7/10	8/10	1/10	0/10
	rHVT-H5(AI)/	2.42 ± 2.60 ^A^	3.69 ± 1.73 ^A^ *	0.00 ± 0.00 ^A^	2.54 ± 1.66 ^B^ *
	rHVT-F(ND)	5/10	8/9	0/10	7/9
7	No vaccine	N.D.	N.D.	N.D.	N.D.
		-	-	-	-
	rHVT-H5(AI)	0.82 ± 1.72 ^A^	1.01 ± 1.80 ^A^	0.00 ± 0.00 ^A^	0.24 ± 0.77 ^A^
		2/10	3/10	0/10	1/10
	rHVT-H5(AI)/	1.09 ± 1.80 ^A^	2.62 ± 2.59 ^A^	0.24 ± 0.74 ^A^	0.00 ± 0.00 ^A^
	rHVT-F(ND)	3/10	5/6	1/10	0/9
10	No vaccine	N.D.	N.D.	N.D.	N.D.
		-	-	-	-
	rHVT-H5(AI)	0.00 ± 0.00 ^A^	0.01 ± 0.04 ^A^	0.00 ± 0.00 ^A^	0.00 ± 0.00 ^A^
		0/10	1/10	0/10	0/10
	rHVT-H5(AI)/	0.44 ± 1.38 ^A^	0.03 ± 0.09 ^A^	0.00 ± 0.00 ^A^	0.00 ± 0.00 ^A^
	rHVT-F(ND)	1/10	1/9	0/10	0/9

^a^ Data presented as mean ± standard deviation of titer equivalent unit value (EID_50_) in milliliters of swabs (log_10_) as determined by QRRT-PCR of 1 mL swabs taken at specific times after challenge. Different uppercase superscript letters (A, B) indicate a significant difference (*p* < 0.05) between vaccination schedules within MDA groups (per column). The asterisk superscript symbol (*) indicates a significant difference (*p* < 0.05) between MDA(–) and MDA(+) groups within vaccination schedule (per line). ^b^ Data presented as frequency (number of virus-positive chickens/total tested chickens). Total numbers of chickens tested were reduced with time because of specific mortality. N.D.: not determined because of specific mortality due to challenge.

**Table 8:** Tracheal and cloacal shedding of H5-HPAIV after challenge at 4 weeks of age with H5-HPAIV to LBL layer chickens (experiment II) vaccinated at day-old with rHVT-F(ND), rHVT-H5(AI), or both vaccines.

Dpi	Vaccine	4 Weeks			
		Tracheal Shedding ^a,b^		Cloacal Shedding ^a,b^	
		MDA(–)	MDA(+)	MDA(–)	MDA(+)
2	No vaccine	8.12 ± 0.75 ^A^ *	5.77 ± 0.90 ^A^	6.30 ± 0.63 ^A^ *	3.48 ± 3.01 ^A^
		10/10	10/10	10/10	6/10
	rHVT-H5(AI)	3.76 ± 2.66 ^B^	3.37 ± 0.62 ^B^	0.46 ± 1.02 ^B^	1.07 ± 1.45 ^A^
		7/10	10/10	2/10	4/10
	rHVT-H5(AI)/	6.23 ± 1.60 ^A^	5.21 ± 1.95 ^A^	1.68 ± 2.76 ^B^	1.80 ± 2.06 ^A^
	rHVT-F(ND)	10/10	9/10	4/10	5/10
4	No vaccine	N.D.	6.99 ± 1.75 ^A^	N.D.	4.61 ± 1.53 ^A^
		-	7/7	-	7/7
	rHVT-H5(AI)	3.40 ± 2.50 ^A^	3.94 ± 1.18 ^B^	0.18 ± 0.38 ^A^	1.40 ± 1.41 ^B^
		7/10	10/10	2/10	6/10
	rHVT-H5(AI)/	3.05 ± 2.83 ^A^	6.51 ± 1.87 ^A^ *	0.00 ± 0.00 ^A^	2.88 ± 2.65 ^AB^ *
	rHVT-F(ND)	6/10	8/8	0/10	5/8
7	No vaccine	N.D.	N.D.	N.D.	N.D.
		-	-	-	-
	rHVT-H5(AI)	0.21 ± 0.64 ^A^	0.14	0.34 ± 0.73 ^A^	0.00
		1/9	1/1	2/9	0/1
	rHVT-H5(AI)/	0.50 ± 1.33 ^A^	3.16 ± 1.89	0.00 ± 0.00 ^A^	0.59 ± 0.83
	rHVT-F(ND)	1/7	2/2	0/7	1/2
10	No vaccine	N.D.	N.D.	N.D.	N.D.
		-	-	-	-
	rHVT-H5(AI)	0.52 ± 1.04 ^A^	0.00	0.00 ± 0.00 ^A^	2.25
		2/9	0/1	0/9	1/1
	rHVT-H5(AI)/	0.00 ± 0.00 ^A^	0.00 ± 0.00	0.00 ± 0.00 ^A^	0.00 ± 0.00
	rHVT-F(ND)	0/7	0/2	0/7	0/2

^a^ Data presented as mean ± standard deviation of titer equivalent unit value (EID_50_) in milliliters of swabs (log_10_) as determined by QRRT-PCR of 1 mL swabs taken at specific times after challenge. Different uppercase superscript letters (A, B) indicate a significant difference (*p* < 0.05) between vaccination schedules within MDA groups (per column). The asterisk superscript symbol (*) indicates a significant difference (*p* < 0.05) between MDA(–) and MDA(+) groups within vaccination schedule (per line). ^b^ Data presented as frequency (number of virus-positive chickens/total tested chickens). Total numbers of chickens tested were reduced with time because of specific mortality. N.D.: not determined because of specific mortality due to challenge.

## Supplementary Materials

The following are available online at https://www.mdpi.com/2076-393X/8/3/536/s1: Figure S1, NDV-specific IgG antibody responses measured in in-house ELISAs-IgG for WL SPF chickens (**a**) and LBL layer chickens (**b**) vaccinated at day-old (experiments I and II, respectively) with rHVT-F(ND) or both rHVT-H5(AI)/rHVT-F(ND) vaccines. Figure S2, AIV-specific IgG antibody responses measured in in-house ELISAs-IgG for WL SPF chickens (**a**) and LBL layer chickens (**b**) vaccinated at day-old (experiments I and II, respectively) with rHVT-H5(AI) or both rHVT-H5(AI)/rHVT-F(ND) vaccines.

## References

[1] Witter R.L., Schat K.A., Saif Y.M. (2003). Marek’s disease. Diseases of Poultry.

[2] Reddy S.M., Izumiya Y., Lupiani B. (2017). Marek’s disease vaccines: Current status, and strategies for improvement and development of vector vaccines. Vet. Microbiol..

[3] Esaki M., Godoy A., Rosenberger J.K., Rosenberger S.C., Gardin Y., Yasuka A., Dorsey K.M. (2013). Protection and antibody response caused by turkey herpesvirus vector Newcastle disease vaccine. Avian Dis..

[4] Palya V., Tatàr-Kis T., Mató T., Felföldi B., Kovács E., Gardin Y. (2014). Onset and long-term duration of immunity provided by a single vaccination with a turkey herpesvirus vector ND vaccine in commercial layers. Vet. Immunol. Immunopathol..

[5] Gardin Y., Palya V., Dorsey K.M., El-Attrache J., Bonfante F., de Wit S., Kapczynski D., Kilany W.H., Rauw F., Steensels M. (2016). Experimental and field results regarding immunity induced by a recombinant turkey herpesvirus H5 vector vaccine against H5N1 and other H5 highly pathogenic avian influenza virus challenges. Avian Dis..

[6] Palya V., Tatár-Kis T., Kovács E.W., Kiss I., Homonnay Z., Gardin Y., Kertesz K., Dan A. (2018). Efficacy of a recombinant turkey herpesvirus AI (H5) vaccine in preventing transmission of heterologous highly pathogenic H5N8 clade 2.3.4.4b challenge virus in commercial broilers and layer pullets. J. Immunol. Res..

[7] Dunn J.R., Dimitrov K.M., Miller P.J., Garcia M., Turner-Alston K., Brown A., Hartman A. (2019). Evaluation of protective efficacy when combining Turkey Herpesvirus–vector vaccines. Avian Dis..

[8] Palya V., Tatar-Kis T., Mato T., Gardin Y. Interference between Different HVT-Vectored Vaccines Applied Subcutaneously at Day-Old in Commercial Broiler. Proceedings of the Annual Meeting of the American Association of Avian Pathologists.

[9] Rauw F., Van Borm S., Welby S., Ngabirano E., Gardin Y., Palya V., Lambrecht B. (2015). Quantification of rHVT-F genome load in feather follicles by specific real-time qPCR as indicator of NDV-specific humoral immunity induced by day-old vaccination in SPF chickens. Avian Pathol..

[10] Rauw F., Palya V., Gardin Y., Tatár-Kis T., Moore Dorsey K., Lambrecht B., van den Berg T. (2012). Efficacy of rHVT-AI vector vaccine in broilers with passive immunity against challenge with two antigenically divergent Egyptian clade 2.2.1 HPAI H5N1 strains. Avian Dis..

[11] Rauw F., Palya V., Van Borm S., Welby S., Tatár-Kis T., Gardin Y., Moore Dorsey K., Aly M.M., Hassan M.K., Soliman M.A. (2011). Further evidence of antigenic drift and protective efficacy afforded by a recombinant HVT-H5 vaccine against challenge with two antigenically divergent Egyptian clade 2.2.1 HPAI H5N1 strains. Vaccine.

[12] Rauw F., Gardin Y., Palya V., van den Berg T., Lambrecht B. (2014). The combination of attenuated ND vaccine with rHVT-ND vaccine at day-old is more protective against NDV challenge than when combined with inactivated ND vaccine. Avian Pathol..

[13] Steensels M., Rauw F., van den Berg T., Marché S., Gardin Y., Palya V., Lambrecht B. (2016). Protection afforded by a rHVT-H5 vaccine against the 2014 European highly pathogenic H5N8 avian influenza strain. Avian Dis..

[14] Van Borm S., Steensels M., Ferreira H.L., Boschmans M., De Vriese J., Lambrecht B., van den Berg T. (2007). An universal avian endogenous real-time reverse transcriptase-polymerase chain reaction control and its application to avian influenza diagnosis and quantification. Avian Dis..

[15] Rauw F., Gardin Y., Palya V., van Borm S., Gonze M., Lemaire S., van den Berg T., Lambrecht B. (2009). Humoral, cell-mediated and mucosal immunity induced by oculo-nasal vaccination of one-day-old SPF and conventional layer chicks with two different live Newcastle Disease vaccines. Vaccine.

[16] Official Journal of the European Union. https://eur-lex.europa.eu/LexUriServ/LexUriServ.do?uri=OJ:L:2009:044:0010:0061:EN:PDF.

[17] Soejoedono R.D., Murtini S., Palya V., Felföldi B., Mató T., Gardin Y. (2012). Efficacy of a recombinant HVT-H5 vaccine against challenge with two genetically divergent indonesian HPAI H5N1 strains. Avian Dis..

[18] Koenen M.E., Boonstra-Blom A.G., Jeurissen S.H.M. (2002). Immunological differences between layer- and broiler-type chickens. Vet. Immunol. Immunopathol..

[19] Chang S., Xie Q., Dunn J.R., Ernst C.W., Song J., Zhang H. (2014). Host genetic resistance to Marek’s disease sustains protective efficacy of herpesvirus of turkey in both experimental and commercial lines of chickens. Vaccine.

[20] Uchida Y., Watanabe C., Takemae N., Hayashi T., Oka T., Ito T., Saito T. (2012). Identification of host genes linked with the survivability of chickens infected with recombinant viruses possessing H5N1 surface antigens from a highly pathogenic avian Influenza virus. J. Virol..

[21] Vervelde L., de Geus E., Jansen C., Heller D.E. (2011). Contribution of the genetic background to the immune response of broilers vaccinated or challenged with LPAI H9N2. BMC Proc..

[22] Rauw F., Gardin Y., Palya V., Anbari S., Lemaire S., Boschmans M., van den Berg T., Lambrecht B. (2010). Improved vaccination against Newcastle disease by an in ovo recombinant HVT-ND combined with an adjuvanted live vaccine at day-old. Vaccine.

[23] Kapczynski D.R., Esaki M., Dorsey K.M., Jiang H., Jackwood M., Moraes M., Gardin Y. (2015). Vaccine protection of chickens against antigenically diverse H5 highly pathogenic avian influenza isolates with a live HVT vector vaccine expressing the influenza hemagglutinin gene derived from a clade 2.2 avian influenza virus. Vaccine.

[24] Tsukamoto K., Saito S., Saeki S., Sato T., Tanimura N., Isoabe T., Mase M., Imada T., Yuasa N., Yamaguchi S. (2002). Complete, long-lasting protection against lethal infectious bursal disease virus challenge by a single vaccination with an avian herpesvirus vector expressing VP2 antigens. J. Virol..

[25] Gao H., Cui H., Cui X., Shi X., Zhao Y., Zhao X., Quan Y., Yan S., Zeng W., Wang Y. (2011). Expression of HA of HPAI H5N1 virus at US2 gene insertion site of turkey herpesvirus induced better protection than that at US10 gene insertion site. PLoS ONE.

[26] Andoh K., Yamazaki K., Honda Y., Honda T. (2017). Turkey herpesvirus with an insertion in the UL3-4 region displays an appropriate balance between growth activity and antibody-eliciting capacity and is suitable for the establishment of a recombinant vaccine. Arch. Virol..

[27] Lardinois A., Vandersleyen O., Steensels M., Desloges N., Mast J., van den Berg T., Lambrecht B. (2016). Stronger interference of Avian Influenza virus-specific than Newcastle Disease virus-specific maternally derived antibodies with a recombinant NDV-H5 vaccine. Avian Dis..

[28] Alexander D.J., Saif Y.M. (2003). Newcastle disease, other avian paramyxoviruses and pneumovirus infections. Diseases of Poultry.

[29] Swayne D.E., Halvorson D.A., Saif Y.M. (2003). Influenza. Poultry Diseases.

[30] Edingert T.O., Pohl M.O., Stertz S. (2014). Entry of influenza A virus: Host factors and antiviral targets. J. Gen. Virol..

[31] Tripathi S., Batra J., Lal S.K. (2015). Interplay between influenza A virus and host factors: Targets for antiviral intervention. Arch. Virol..

[32] Ganar K., Das M., Sinha S., Kumar S. (2014). Newcastle disease virus: Current status and our understanding. Virus Res..

[33] Jardetsky T.S., Lamb R.A. (2014). Activation of paramyxovirus membrane fusion and virus entry. Curr. Opin. Virol..

[34] Smith E.C., Popa A., Chang A., Masante C., Dutch R.E. (2009). Viral entry mechanisms: The increasing diversity of paramyxovirus entry. FEBS J..

[35] Morgan R.W., Gelb Jr J., Schreurs C.S., Lütticken D., Rosenberger J.K., Sondermeijer P.J. (1992). Protection of chickens from Newcastle and Marek’s diseases with a recombinant herpesvirus of turkeys vaccine expressing the Newcastle disease virus fusion protein. Avian Dis..

[36] Hasselquist D., Nilsson J.-A. (2009). Maternal transfer of antibodies in vertebrates: Trans-generational effects on offspring immunity. Philos. Trans. R. Soc. Lond. B Biol. Sci..

[37] Carlier Y., Truyens C. (1995). Influence of maternal infection on offspring resistance towards parasites. Parasitol. Today.

[38] Watts C., Antoniou A., Manoury B., Hewitt E.W., Mckay L.M., Grayson L., Fairweather N.F., Emsley P., Isaacs N., Simitsek P.D. (1998). Modulation by epitope-specific antibodies of class II MHC-restricted presentation of the tetanus toxin antigen. Immunol. Rev..

[39] Glezen W.P. (2003). Effect of maternal antibodies on the infant immune response. Vaccine.

[40] Siegrist C.A. (2003). Mechanisms by which maternal antibodies influence infant vaccine responses: Review of hypotheses and definition of main determinants. Vaccine.

[41] Ingrao F., Rauw F., van den Berg T., Lambrecht B. (2017). Characterization of two recombinant HVT-IBD vaccines by VP2 insert detection and cell-mediated immunity after vaccination of specific pathogen-free chickens. Avian Pathol..

[42] Ingrao F., Rauw F., Steensels M., van den Berg T., Lambrecht B. (2018). Early immune responses and profiling of cell-mediated immunity associated gene expression in response to rHVT-IBD vaccination. Vaccine.

