# Supplementary material for: Effectiveness of a Simultaneous rHVT-F(ND) and rHVT-H5(AI) Vaccination of Day-Old Chickens and the Influence of NDV- and AIV-Specific MDA on Immune Response and Conferred Protection
Source: Vaccines (Basel). 2020 Sep 16;8(3):536. doi: 10.3390/vaccines8030536 (PMC7565404; doi:10.3390/vaccines8030536)
Supplement: Supplementary file 1 [file vaccines-08-00536-s001.pdf]

vaccines

Supplementary Materials: The following are available online at www.mdpi.com/xxx/sx :

Figure S1: Title "NDV-specific IgG antibody responses measured in in-house ELISAs-IgG for WL SPF chickens (**a**) and LBL layer chickens (**b**) vaccinated at day-old (experiments I and II, respectively) with rHVT-F(ND) or both rHVT-H5(AI)/rHVT-F(ND) vaccines"

Figure S2, Title "AIV-specific IgG antibody responses measured in in-house ELISAs-IgG for WL SPF chickens (**a**) and LBL layer chickens (**b**) vaccinated at day-old (experiments I and II, respectively) with rHVT-H5(AI) or both rHVT-H5(AI)/rHVT-F(ND) vaccines"

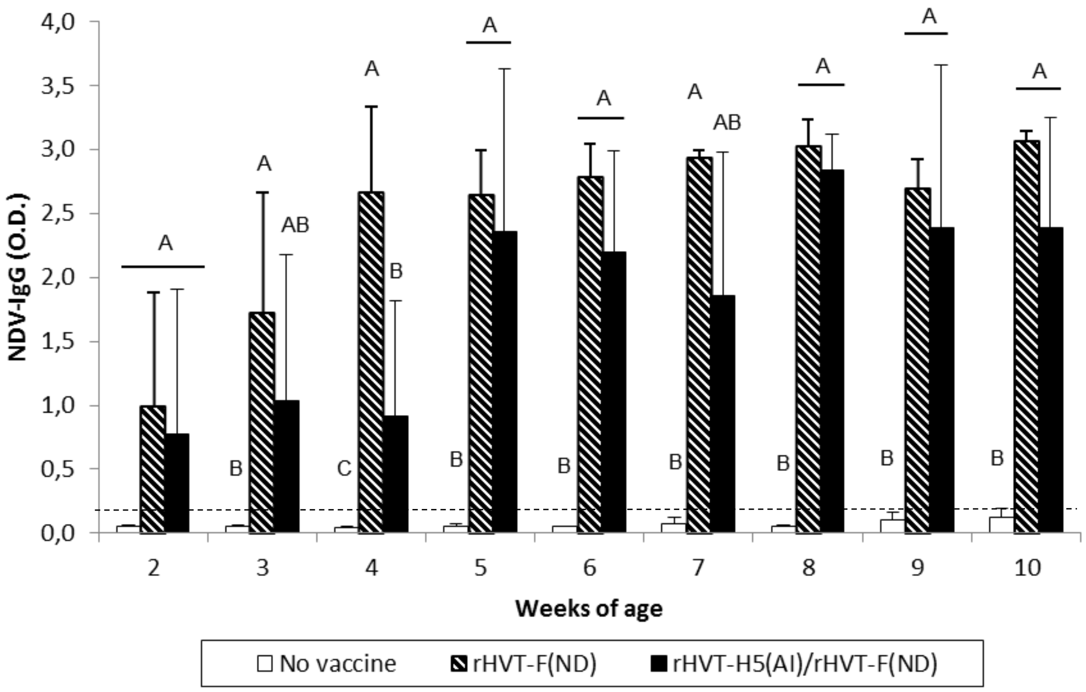

(**a**)

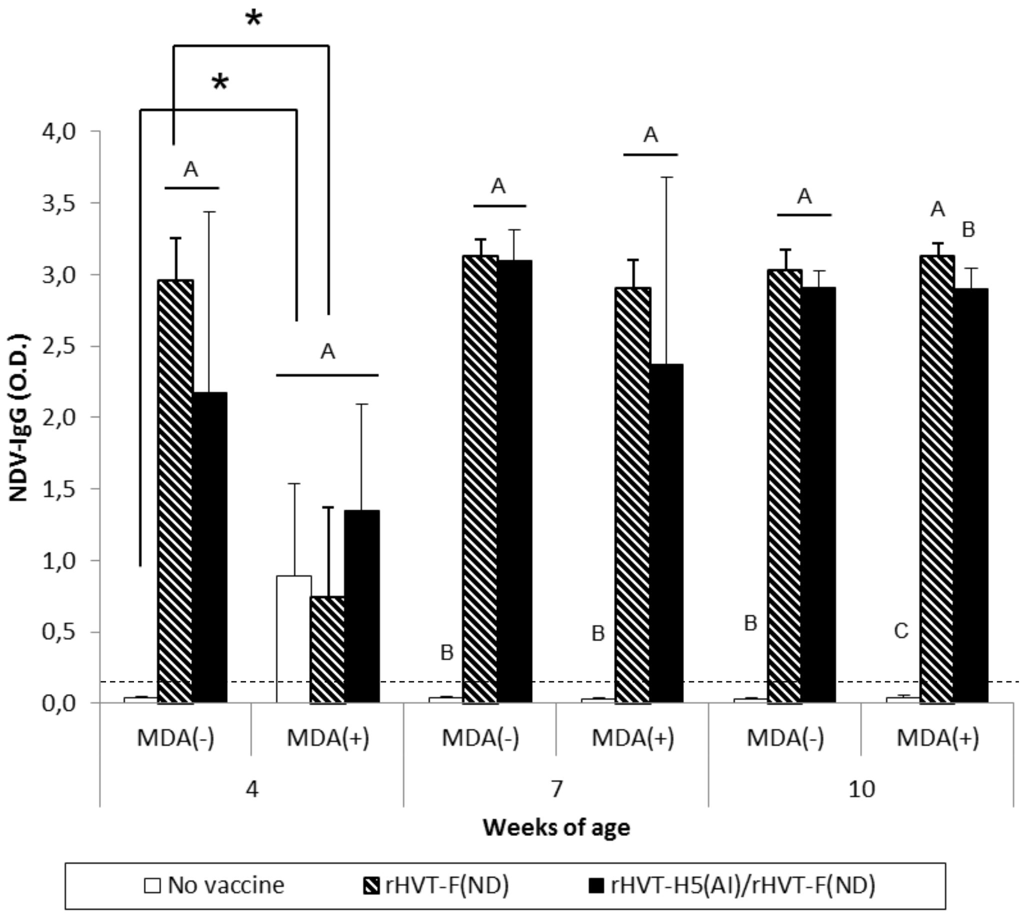

(**b**)

Figure S1. NDV-specific IgG antibody responses measured in *in-house* ELISAs-IgG for WL SPF chickens (**a**) and LBL layer chickens (**b**) vaccinated at day-old (experiments I and II, respectively) with rHVT-F(ND) or both rHVT-H5(AI)/rHVT-F(ND) vaccines. Data represent mean ± standard deviation

of absorbance values by ELISA at specified ages (n = 5). Serum samples were diluted 1:400. Optical density (O.D.) values ≥ 0.100 were considered positive (this threshold of positivity is indicated by the dotted line and was set at [average + 2 x standard deviation of sera from SPF chickens]. In figure **a**, mean ± standard deviation at time points with no common uppercase letters indicates a significant difference between vaccination schedules ($P < 0.05$). In figure **b**, mean ± standard deviation at time points with no common uppercase letters indicates a significant difference between vaccination schedules within the MDA group ($P < 0.05$), while (*) indicates a significant difference between MDA(-) and MDA(+) groups within a given vaccination schedule ($P < 0.05$).

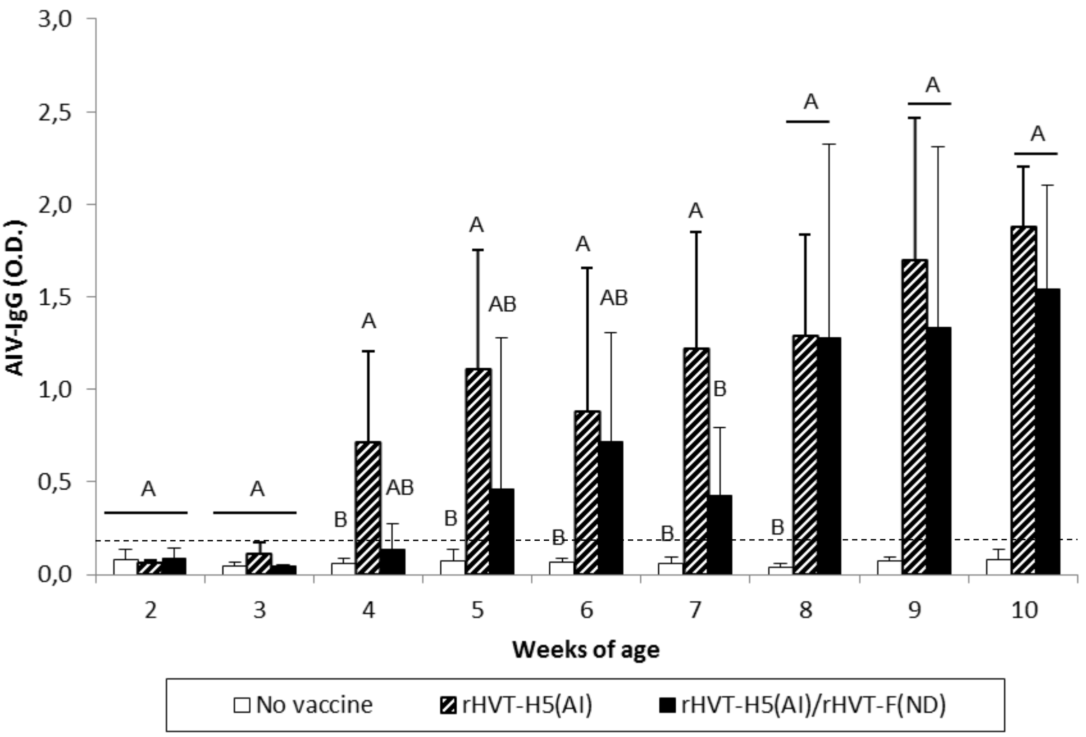

(**a**)

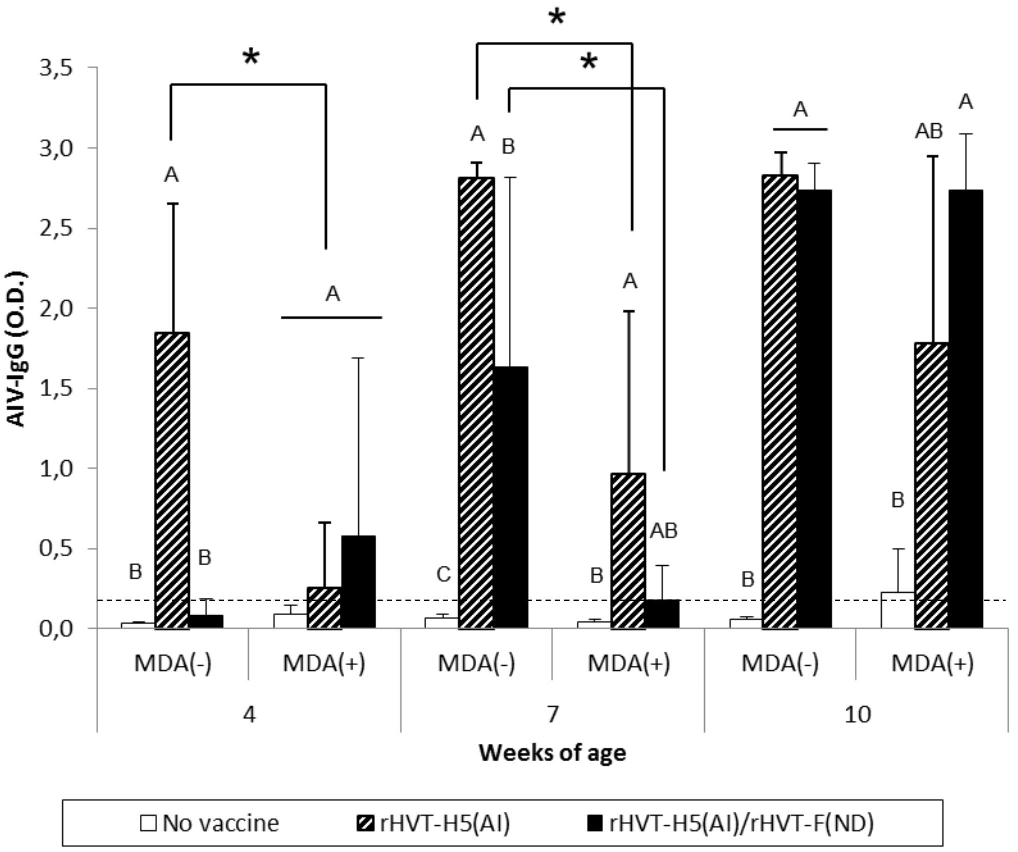

(**b**)

Figure S2. AIV-specific IgG antibody responses measured in *in-house* ELISAs-IgG for WL SPF chickens (**a**) and LBL layer chickens (**b**) vaccinated at day-old (experiments I and II, respectively) with rHVT-H5(AI) or both rHVT-H5(AI)/rHVT-F(ND) vaccines. Data represent mean ± standard deviation

of absorbance values by ELISA at specified ages (n = 5). Serum samples were diluted 1:100 or 1:200 (experiment I and II, respectively). Optical density (O.D.) values ≥ 0.100 were considered positive (this threshold of positivity is indicated by the dotted line and was set at [average + 2 x standard deviation of sera from SPF chickens]. In figure **a**, mean ± standard deviation at time points with no common uppercase letters indicates a significant difference between vaccination schedules ($P < 0.05$). In figure **b**, mean ± standard deviation at time points with no common uppercase letters indicates a significant difference between vaccination schedules within the MDA group ($P < 0.05$), while (*) indicates a significant difference between MDA(-) and MDA(+) groups within a given vaccination schedule ($P < 0.05$).

© 2020 by the authors. Submitted for possible open access publication under the terms and conditions of the Creative Commons Attribution (CC BY) license (http://creativecommons.org/licenses/by/4.0/).
